# Quantitative MR-neurographic parameters can determine and specify nerve injury in amyloid related polyneuropathy

**DOI:** 10.1186/1750-1172-10-S1-O14

**Published:** 2015-11-02

**Authors:** Jennifer Kollmer, Ernst Hund, Stefan Schönland, Ute Hegenbart, Christoph Kimmich, Arnt Kristen, Martin Bendszus, Mirko Pham

**Affiliations:** 1 University of Heidelberg, Department of Neuroradiology, 69120, Heidelberg, Germany; 2 University of Heidelberg, Department of Neurology, 69120, Heidelberg, Germany; 3 University of Heidelberg, Medical Department V (Amyloidosis Center), 69120, Heidelberg, Germany; 4 University of Heidelberg, Medical Department III (Amyloidosis Center), 69120, Heidelberg, Germany

## Background

Hereditary transthyretin-familial-amyloid-polyneuropathy (TTR-FAP) usually manifests with a rapidly progressive, distally-symmetric polyneuropathy [Plante-Bordeneuve, V. and G. Said, Lancet Neurol, 2011; Hund et al, Neurology 2001]. Recently, we were able to show that nerve-injury in TTR-FAP is detectable in-vivo by applying high-resolution MR-Neurography [Kollmer et al, Brain 2015]. The aim of the current study is to further quantify nerve-lesions at thigh-level where nerve-injury has been shown to be strongest, and to determine the ability of two quantitative parameters to clearly differentiate between symptomatic TTR-FAP, asymptomatic gene-carriers and healthy volunteers.

## Methods

20 patients with confirmed mutations in the TTR-gene (13 with symptomatic TTR-FAP, 7 asymptomatic gene-carriers), and 40 age/gender-matched healthy volunteers were prospectively included and classified according to neurological and electrophysiological findings. MR-Neurography with high structural resolution was performed on a 3T-MR-scanner (Magnetom/TIM-TRIO/Siemens):1) T2-TSE-fs (TR/TE 5970/55ms, voxel-size 0.4x0.3x3.5mm^3^); 2) Dual-echo-TSE-fs (TR 5210ms, TE1/TE2 12/73ms, voxel-size 0.4x0.3x3.5 mm^3^).

Manual voxel-vise segmentation of the sciatic/tibial/common-peroneal nerve with subsequent fully-automatic classification as nerve-lesion-voxels was performed on each axial imaging slice (280/subject). The apparent-T2-relaxation-time (T2app) and proton-spin-density as distinct and quantifiable parameters that measure microstructural nerve-tissue-composition in-vivo [Heiland et al, Neurosci Lett. 2002] were then calculated for all nerve-lesion-voxels.

## Results

One-way-ANOVA and post-hoc comparisons showed that proton-spin-density was highest in symptomatic TTR-FAP (549.97±35.78), decreased significantly in asymptomatic gene-carriers (406.09±28.22; p=0.002), and further decreased significantly in controls (286.56±10.04; p<0.0001 vs. symptomatic TTR-FAP and vs. asymptomatic gene-carriers (p=0.004).

Post-hoc comparisons showed that T2app was significantly increased only in symptomatic TTR-FAP (103.92ms±6.4) vs. asymptomatic gene-carriers (79.14ms±1.8; p=0.012) and vs. controls (84.08ms±2.54; p=0.003), but not between asymptomatic gene-carriers and controls (p=0.783).

## Conclusion

For the first time, we were able to prove that alterations of the evaluated quantitative markers were highly specific: Asymptomatic carrier-status and symptomatic disease were both closely associated with a strong increase of proton-spin-density, while a significant increase of the T2-relaxation-time was found only in symptomatic TTR-FAP, but not in asymptomatic carriers. These findings suggest that proton-spin-density is more sensitive for the detection of early or even subclinical nerve-lesions, while T2app may serve to specifically differentiate increasing disease severity in already symptomatic TTR-FAP.

